# The Role of Women on Dairy Goat Farms in Southern Spain

**DOI:** 10.3390/ani12131686

**Published:** 2022-06-30

**Authors:** Cristina Arce, Cipriano Díaz-Gaona, Manuel Sánchez-Rodríguez, Santos Sanz-Fernández, Mª. Dolores López-Fariña, Vicente Rodríguez-Estévez

**Affiliations:** 1 Department of Animal Production, Campus Rabanales, University of Córdoba, 14014 Córdoba, Spain; pa2digac@uco.es (C.D.-G.); pa1sarom@uco.es (M.S.-R.); v22safes@uco.es (S.S.-F.); pa2roesv@uco.es (V.R.-E.); 2 ACRIFLOR (National Association of Florida Goats Breeders), Campus Rabanales, University of Córdoba, 14014 Córdoba, Spain; mdoloresperez@acriflor.org

**Keywords:** gender, productivity, Florida breed, benchmarking, family farms, sustainability

## Abstract

**Simple Summary:**

The involvement of women in livestock production is a tradition around the world, where women have been associated with small animal farming, mainly based on family production systems. They used to be responsible for animal care (feeding and milking) and family care (children and the elderly), with the working hours exceeding the daily working time defined in developed countries. Men are responsible for the farm’s management and finances. In Europe, small ruminants are mainly raised in adverse environments. Nevertheless, they contribute to maintaining an open environment and the prevention of fire damage in times of drought. On the farms studied, a lower incidence of mastitis in goats was found when a woman worked on the farm (they milk with more care and patience). In this study, there is a predominance of farms with women and men (a family unit), where the genders both equally participate when making decisions that involve household expenses; however, this shared control usually ends up being transferred to the man. In addition, very few women are the owners of farms or make binding decisions. Finally, the inclusion of women should be recommended throughout the small ruminant productive process: this would help improve farming results and provide an incentive to keep women in rural areas.

**Abstract:**

One of the factors involved in goat milk production is the role of women as farmers. The aim of this study was to evaluate the role of women on dairy goat farms, considering: (1) the profile of women occupationally involved, (2) the organization of the women’s work, (3) the degree of involvement by women in the decision-making on these farms, and (4) the influence of women’s work on productive results. This study was conducted on 52 dairy goat farms in southern Spain. A descriptive analysis and means comparisons were performed to describe the farms where any women were involved or not. In 61.5% of the farms, at least one woman was involved, with an age of 42.2 ± 8.8 years. Very few women were farm owners, although women took binding decisions in 81.25% of these farms. Their work is dedicated to milking and caring for the kids. Women had a positive influence on the productive variables analysed, and for mastitis in herds, the incidence was lower in herds where women participated (*p* < 0.01). In conclusion, it is recommended to include women’s work as a factor when characterizing dairy goats farms’ systems to evaluate their positive effect on a farm’s performance.

## 1. Introduction

A total of 37.2% of the Spanish goat population is located in Andalusia, and it produces 42.1% of Spain’s goat’s milk [1]. However, in this region and in the rest of Spain, goat production systems are in a critical situation, basically due to the increase in production costs [2]. In the last two decades, this sector has improved rapidly, resulting in a specialization in milk production. The predominating breeds that specialize in high milk production, such as the Florida, Malagueña, Murciano-Granadina and Majorera breeds in southern Spain, are mainly based on a family production system. They are mostly free range without grazing, medium size (about 400 breeding females) and are family farms [3]. Moreover, like in other countries, livestock rearing has become a major livelihood strategy among rural farmers, especially for smallholders and marginal farmers [4]. In marginal rural areas, where poverty is rampant, livestock represents an important asset for the local cultural and socio-economic systems and allows the effective use of otherwise non-utilizable resources [5,6].

For the foregoing, in order to make a proper analysis of these production systems, it is important to efficiently evaluate the participation of all the factors involved in the production process. One of these factors is the role of women as food providers and producers; in addition, their wide experience in rural environments makes them unique knowledge carriers and decision-makers on local species and ecosystems [7,8]. In fact, it is well-documented that sheep, goats and their products are important tools to improve the living conditions of poor women [5,8]. Moreover, small ruminants are a means of opening the door for women to microeconomic business, and grants them the opportunity to contribute to their family’s food security [8,9].

In the recent past, some researchers have focused their studies on the relationship between gender and livestock production [10,11,12]. Nevertheless, in this regard, Kristjanson et al. [11] highlighted the limited research done recently on the role of rural women in livestock farming, which has limited the opportunities offered due to specific interventions and public policy. Hence, it is important to keep in mind that the gender responsible for the small ruminant production system varies from region to region and depends upon the family type, culture, religion, stage of economic development, species of predominant animals reared and population pressure [4]. For example, in the transhumant Peul society, men tend to own mainly cows and camels, and women own goats, sheep and poultry [13]. In contrast, there is extensive research about the role of women in agriculture, especially in small-scale cultures, where their importance is widely proven. On the other hand, there are few studies comparing the productivity of the livestock managed by women versus the livestock managed by men [10]. Their roles vary considerably between and within regions and are changing rapidly in many parts of the world [4].

Thus, in countries such as Najran (Arabia Saudi), a study by Aldosari [14] revealed significant differences between the two genders: male herders were more experienced, received more benefits, showed greater interest in discussions on topics related to sheep and goat farming, followed more information from radio and TV, and received more services offered by veterinary clinics. Female herders, however, received fewer services, and the veterinary clinics proved to be less beneficial for them. In rural regions of Turkey, where small ruminant farming plays an important social role, the distribution of the work was unfavourable to women, with their position in decision-making processes being secondary [15]. On the contrary, Verbeek et al. [16] suggested that women who work in goat-intensive small farms in Kenya participated in a greater proportion of the herd’s reproductive management compared with women who work in goat-extensive systems. Similarly, Betty [17] indicated that there is a male dominance in cattle disposition and control, regardless of whether the owner of the farm is male or female, indicating that only in 3% of the farms operated by men are women allowed to control and derive benefits from the cattle. In any case, it would be necessary to study the performance of rural women’s farm activities and decision-making to evaluate the convenience or not of their implication in these production systems.

In rural Spain, women have traditionally worked in activities related to agriculture and livestock [9,18]. In this sense, small ruminants can be tethered near the home and along community access routes, circumventing the husbandry restrictions imposed on many women [9]. The predominant rural productive activity in Spain is developed through the family farm; therefore, it is a family business based on the couple working together, where the woman often helps her husband in many everyday tasks. However, this work tends to be of a complementary nature, in which a woman’s presence and work are essential for the maintenance of her family and the farm [19]. Nevertheless, the diagnoses of the Spanish rural countryside continue to remark on the fragility of female employment in these areas [8,19,20]. Merino [21] refers to the family support provided by the women on Spanish farms, indicating that, on 82% of the farms, women are considered domestic help. To understand the fragility of the rural sector, it is crucial to understand the gender structure, with a special emphasis on the organization of the women’s daily life [22,23]. Camarero et al. [22] indicated the need for further research on gender equality in Spanish rural environments and the mechanisms to ensure the stable retention of women in these conditions. Hence, the knowledge of the involvement of women in these herds is essential to develop tools that can be transferred to the sector and assist in the upkeep of these farms [3,24,25,26]. It has been considered that the profile of women as livestock farmers can be defined from various perspectives: family relationships with the owner, and the type of activity and age [27]. However, women in rural areas perform various roles: from managing the household chores to taking care of the children and livestock.

The aim of this study is to evaluate the role of women in dairy goat farms in Andalusia (southern Spain), considering:

1. The profile of women occupationally involved on dairy goat farms.
2. The organization of women’s work on these farms.
3. The degree of involvement of women in decision-making on these livestock farms.
4. The influence of women’s work on productive results.

## 2. Materials and Methods

### 2.1. Sampling

This study was conducted in collaboration with the Association of Florida Breed Farmers (ACRIFLOR) on farms located in the south of Spain, a region with a Mediterranean climate. The sample comprised 52 farms (92.9% of the associated goat farms). A survey (Supplementary Materials) for data collection was designed: this covered social, technical and economic information and was structured on the basis of the following sections:

- Goat breeder or goat herder woman’s profile (age, number of children and age, years of experience in goat rearing, education, ownership).
- Work and task distribution.
- Decision-making responsibility.
- Productive parameters and performance of the dairy goat farm.

The information was gathered through direct interviews with the producer, and it was completed with the collection and processing of production data recorded in the ACRIFLOR database.

### 2.2. Statistical Analysis

For the overall analysis of the data, SPSS^®^ 12.0 (IBM SPSS, Chicago, IL, USA), was used. The software was sourced by the University of Córdoba, Spain. Descriptive statistics were analysed for the quantitative variables using measures of dispersion and position, and Chi-square was used as the test of significance for the qualitative variables. An independent samples t-test for the mean comparison was applied to analyse how the presence of women affects the productive results.

## 3. Results and Discussion

### 3.1. Dairy Goat Farm Characteristics

In the herds studied, the average size was 401.9 ± 269.2 for adult animals, with 395.5 ± 252.6 for breeding goats and an average milk production of 1.82 ± 0.44 kg/goat/day and 423 ± 132.7 kg/goat/year. The farms studied were characterized by a slight predominance of the fully-stabled (53.8%) versus semi-extensive systems (46.2%), according to the Sánchez-Rodríguez classification [3].

Among the semi-extensive farms, 46.2% of these were linked to any agroecosystem: they were mainly linked to dehesas (50%) and were in second place to the agricultural countryside (29.2%), with the seasonal use of crop residues. A total of 12.5% of herds grazed scrubs and bushes in the mountains, while a smaller proportion used olive groves (8.33%). In this regard, it has been quoted that there is a wide diversity among southern Spanish dairy goat farms, which are divided into two systems types: one in which animals are fully stabled, and the others, where animals graze under different time modes and plant resources [3,24].

With respect to the business organization, the family-type company was the most prevalent (71.2%), followed by the individual company (21.2%) and finally, the society company (7.7%). Other researchers have already shown this, highlighting the prevalence of some common features in southern Spain, such as the family nature of the farm [25,28,29]. There were 32 farms (61.53%) where at least one woman worked. A comparison between the arms with or without farmer women is shown in Table 1.

### 3.2. Women’s Profile on Dairy Goat Farms

The average age of women livestock farmers was 42.2 ± 8.8 years, with 10.4 ± 8.5 years of experience working with goats (Table 2). A similar average profile was found on the Swaziland and Tirupati goat farms, where 41.4% of women were aged between 41 and 50 years [30], and in the Bijapur district of the Karnataka goat farms, where 65% of the small ruminant farmers were middle-aged [31]. Merino [21] showed 50 years old as the mean age for the Spanish rural women, which could be explained as a return to farming after a time with the prevalence of the migration of younger and better-educated women towards urban areas in search of alternative employment opportunities [32,33].

As described by Mabe et al. [34], the age factor influences the development of goat-breeding activities. These researchers indicate that women over 50 years old have limited physical abilities that are important for livestock management. Moreover, this age can affect the decision-making processes necessary for innovation in the production unit, while younger farmer women tend to take more risks and, therefore, are more likely to incorporate new technologies on the farm, as dairy and intensive farms require.

The predominating woman represents what Camarero et al. [22] defined as “generational support” in the Spanish countryside, i.e., the woman who supports the majority of the dependent population, such as children, adolescents and the elderly. By contrast, there was a low proportion of childbearing-age women (Table 2) because 87.5% of the women working on the studied farms had children, with an average age of 15.3 ± 8.4 years (Table 2). Similar results were found by Camarero et al. [22], who observed a lower proportion (18.2% of the population) of women of reproductive age (considered as 24–34 years old), which may be a limiting factor for the permanence of these production systems. This lack of women in some of these strategic years has been pointed out as having an important impact on the social sustainability of rural communities [22].

Most goat herder women had some level of education (92.6%); 71.9% of these had completed their primary education, and a low proportion had finished their secondary education or had university degrees (Table 3). Similar values of age, education and family size have been reported by other researchers [32,33,34]. Fernández-Sanchidrián [35] shows a lower percentage of education for rural women from Valladolid (central Spain), where only 50.8% of the women completed their primary education. Hence, in spite of the low levels of education found, these results show goat herder women with a higher educational level, mainly when compared to other countries; i.e., 55.2% of the women had primary studies on goat farms in Swaziland [30]; 38.3% illiteracy was described by Channappagouda [31]. In this regard, it is noted that education is generally regarded as an important variable that can enhance the adoption of new technologies [36,37]; however, it can also be considered a cause of rural abandonment and the uprooting of field activities [22].

Table 3 shows the characteristics that sum up the education and owner relationship of the women involved in the production process of the farms studied. Only 9.4% of these women were not related to the male owners, while 71.9% were married to them. These results are similar to those shown by Martín et al. [38], who pointed out that on Spanish organic farms, the number of women heads of the farms was low, whether they worked on the same farm or not. These results agree with the research from developing countries, where women generally do not have ownership of the land, nor the management of it [16,31]. Camarero et al. [22] indicated that, although many women are the owners of property, they find it difficult to assume this role in the same way as a man. In fact, in the current study, there are no women owners.

Only 21.9% of women received a salary of an average amount of EUR 900 per month, which exceeds the Spanish minimum salary of EUR 735.9 per month [39]. However, 78.1% of the women goat-herders are not remunerated for their work, suggesting that most of them have no economic independence (Table 3). This aspect was already pointed out by Camarero et al. [22], who suggested that if women receive a payment for the work that they carry out, it could be a way to reach their personal autonomy. It is estimated that in Spain, there are around one million women working on family farms, which is not reflected in the statistics; this is the so-called “family assistance” or “assisting spouses” [40]. Many of these activities are not defined as “economically active employments” in the national labor force survey, but they are essential to the well-being of rural households. Despite a woman´s involvement in the day-to-day care, livestock management is still considered a man’s role by livestock managers and decision-makers because the work that women do is seldom recognized [41].

Hence, there is a lack of gender equity, and according to Gustafson et al. [42], this equity is one of the four “sociocultural wellbeing” indicators, which in turn is one of the Seven Food System Metrics of Sustainable Nutrition Security proposed by them.

### 3.3. Division of Work on Dairy Goat Production Units

Women working in these production units totalled an average of 4.9 ± 2.9 h/day (Table 4). This value is lower than that found in other Mediterranean countries, i.e., Turkey, where women working on small ruminant farms devote 12.6 h/day to working with animals, mainly grazing them [15]. However, as has been mentioned previously, only 46.2% of the farms studied (the semi-extensive ones) had grazing activity; and this exclusively corresponded to men. Additionally, all women did housework (spending an average of 5.8 ± 2.1 h/day) (Table 4). In this regard, Merino [21] found that the Spanish rural women worked an average of 8 h at home. Moreover, these data agree with Camarero et al. [22], who indicated the double-working day (housework and livestock care) of women in Spanish rural areas. This amount may involve a significant reduction in recreation time or other activities outside the farm and home. However, this critical role assumed by women is key to attaining each of the pillars of food security (availability, access and utilization) [42].

Women spent long-time milking (Table 4), which means that most of the women’s farm-working day is occupied with milking and kid care (1.8 ± 1.1 vs. 1.7 ± 2.0 h/day, respectively). In France, most women are mainly responsible for milking and caring for the calves, while in Uruguay, they are involved in these activities just as helpers [23].

Likewise, in the Villuercas-Ibores area (southwest of Spain), the semi-extensive goat farms mainly use family labour, principally constituted by a full-time worker, which is usually the owner, and a part-time worker, which is usually the owner’s wife or son [25]. Acosta et al. [43] indicated that during the first two-thirds of the twentieth century, in the dehesa of Extremadura (southwest of Spain), goat-herding women were responsible for making cheese, plus some other temporary assignments, while always being responsible for making cheese for self-consumption. According to these results and previous studies [23,25], milking and kid care could be considered the duty of dairy farmer women in developed countries.

The age of the children does not affect the hours of farm work, with an average of 4.0 ± 2.6 h for mothers with children aged <10 years and 5.2 ± 3.2 h for those with children older than 10 years (*p* > 0.05). Different results have been reported by other researchers, who suggest that it is possible that women with young children have limited participation in the work on farms. In this regard, Chiappe [44] concluded that a child’s age directly affects a woman’s participation in farm activities.

### 3.4. Women’s Satisfaction with Livestock Working

A total of 71.9% of the women working on the farms studied, who were relatives of the farmers, did not want to do another activity (Table 5), especially women who were farmer sisters or mothers (*p* < 0.05): they felt attached to the goat farm.

### 3.5. Participation of Women in Decision-Making

A total of 81.3% of farmer women made different handling decisions on the farms. However, the sale of either animals or milk was primarily an activity coordinated by men; only in 26.7% of the farms did women make binding decisions in the marketing process (Table 6). In this regard, on the farms of the Turkish Association of Mohair, women play an important role in deciding aspects of goat management, such as the time of weaning, feeding, and milking time. However, the men decide everything related to the sales and investments, and they are responsible for farm planning and organization [45].

This highlights the fact that, among the farms studied, the predominance corresponded to those in which women and men did not participate equally in decisions involving loan applications and household costs; emphasizing that the role of women was relevant and more participative when compared to studies in developing countries [45]. This feature is of great social significance because it has been confirmed that when women have more influence over economic decisions, their families spend more income on food, health, education, clothing and children’s nutrition [46]. For these reasons, the social safety-net programs in many countries are now focusing on women [46] and have produced guidelines for the best way to support these women [47].

On French dairy cattle farms, two kinds of decisions were found: (i) economic and financial decisions directly related to the handling of money or capital, and (ii) technical decisions involving technological innovations or the technical management of the farm. Although women were responsible for the accounting and, therefore, they well understood the economic and financial aspects of the operation on these farms, the first group of decisions was usually taken by their husbands [23].

The farms studied presented a partial self-consumption, mainly characterized by the separation of milk that was used to make cheese for home consumption (this was done on 59.4% of the farms where a woman was involved); thus, the decision to divert milk to make cheese was made by women on 30.8% of the farms (Table 6). This coincides with the results of Guadalajara-Olmeda et al. [27] on farms in Spain and Italy, although they found greater participation of women in deciding the milk consumption on the farm. In this regard, recent research associates women with small animal farming, such as chickens, sheep and goats, more than with large animals, and they are mainly responsible for animal care (feeding, water supply, cleaning barns and milking) [11,34,48].

In many countries, the “patriarchal agrarian systems” continue to dominate agriculture, in which both the men and women make up the workforce; however, it is the men who control the decision power and production results [49]. In some societies, especially in developing countries, women control the milk production when its use is for home consumption, but they cannot sell it and manage its income [50]. Moreover, they show a great increase in the world’s food security by improving the health and livelihood of individual families [48].

### 3.6. Influence of Women’s Work on the Productive Results of Farms

On the farms studied, a lower incidence was found of mastitis in goats when a woman worked, compared to farms without any women (4.7 ± 3.5 vs. 9.1 ± 7.6 goats with mastitis problems, respectively, representing 1.2% and 2.0% of the goats) (*p* < 0.01). According to Courdin et al. [23], these results are justified by the fact that women milk with more care and patience, being why they are selected for this activity. Some studies highlight the essential female gender characteristics to perform these tasks, such as patience and dedication. This affects the type of tasks they perform every day with the animals, although there is a lack of appreciation for their role on the farm [23]. Moreover, an improvement in udder health would be expected after some training. In this regard, on buffalo farms in Nepal, a significant positive effect was found in training women on the lower prevalence of mastitis on these farms compared to farms where women had no training [50]. In addition, according to FAO [51], a rural woman’s access to agricultural extension services worldwide is only about one-twentieth of that of men.

With respect to kid mortality, there were no significant differences between farms; however, a tendency was observed towards a lower kid mortality on farms where there were women working (6.1 ± 4.5 vs. 7.4 ± 6.2%). Moreover, an important tendency has been found to implement reproductive techniques where there were women working (62.5 vs. 37.5% of farms).

Ragasa et al. [52] and Sabo [37] agree that the incorporation of women into a farm’s productive work has a positive impact, taking into consideration that with their important participation, the benefits are greater, especially in terms of sustainability in Nigeria. The study of Sabo [36] found that a woman’s participation in a training program showed a great improvement in agricultural production on 78% of the farms, a slight improvement on 20%, and there was none on 2% of the farms; concluding that the training of women has positive effects on the productive results of the farms where they work. On French dairy cattle farms, it was found that female farmers were associated with more positive beliefs about the importance of contact and having interactions with calves; moreover, female farmers were observed as having more positive contact with the calves, and this can result from the more positive attitude they hold towards them [53].

Finally, according to Fernández-Jiménez et al. [8], it is important to create women’s networks to address their specific needs to advance rural sustainability, consider their priorities and increase their public visibility in pursuing social and economic change.

## 4. Conclusions

Women are an important component in dairy goat production systems.

1. Among the women working on goat farms, those who represent important generational support for the care of the family’s children and elderly predominate. The implication of women in both agricultural activities and housework makes a woman’s working hours exceed the daily working time considered for welfare in developed countries.
2. The in-depth study of their role is essential to understand both the farms and family management because the family unit develops strong ties of economic and productive cooperation, which is very important for small dairy farm units.
3. On the farms studied, those where women and men who compose the family unit and participate equally when making decisions that involve borrowing and household expenses, predominate. However, very few women are owners of the farms or make binding decisions in the marketing process; and commercialization, formerly under women’s control or participation, often ends up with a transfer of control to men. This is a significant event that characterizes Spanish rural society.
4. A significant positive influence of women on the productive variables analysed is detected on the farms studied, where much of a woman’s working day with the animals is dedicated to milking and caring for kids, which seems to affect the productive results obtained positively.

Finally, the inclusion of women’s work evaluations should be recommended as a benchmarking factor when characterizing dairy goat farming systems throughout the production process. This would facilitate a better knowledge of these systems and their performance, and it would help to design human resource strategies as well as to improve farm management organization.

## Figures and Tables

**Table 1:** Characteristics of farm with or without farmer women (mean ± SD).

	Farm with Farmer Women	Farm without Farmer Women
Intensive system (*n*)	22	6
Semi-extensive system (*n*)	10	14
Family-type company (*n*)	26	11
Individual company (*n*)	4	7
Society company (*n*)	2	2
Goats (mean ± SD)	449.20 ± 303.21	372.28 ± 245.98
kg milk/goat/day (mean ± SD)	1.86 ± 0.49	1.75 ± 0.38

SD: Standard deviation.

**Table 2:** Characteristics of the profile of livestock farmer women (mean ± SD).

Characteristics of Women	Mean ± SD	Minimum	Maximum
Woman’s age (years)	42.19 ± 8.78	21	53
Experience with goats (years)	10.44 ± 8.52	1	35
Children (*n*)	1.97 ± 1.05	0	4
Children age (years)	15.31 ± 8.35	1	36

SD: Standard deviation.

**Table 3:** Characteristics of the profile of livestock farmer women.

Characteristics of the Profile of Women Livestock Farmer	*n*	Distribution (%)
Woman Education Level (Highest Level Completed)		
Without studies	3	9.4
Primary school	23	71.9
Secondary or high school	3	9.4
University studies	3	9.4
**Family Relationship between Farmer Woman and the Owner**		
Wife	23	71.9
Sister	4	12.5
Mother	2	6.3
Without relationship	3	9.4
**Salary**		
Without salary	25	78.1
With salary	7	21.9

**Table 4:** Daily work distribution of women dairy goat farmers (hours).

Women’s Work (Hours)	Mean ± SD (%)	Minimum	Maximum
At home	5.80 ± 2.06 (45.8)	2	8
On farm	4.86 ± 2.92 (54.2)	1	12
Total	5.2 ± 2.76		
**Daily Work Distribution of Women in Dairy Goat Farms (Hours)**			
Milking	1.84 ± 1.09 (36.1)	1	4
Kids care and goat labour	1.71 ± 2.04 (33.5)	1	7
Cheese-making	0.67 ± 1.27 (12.7)	2	4
Herd-feeding	0.41 ± 0.63 (7.6)	1	2
Facility-cleaning	0.40 ± 0.56 (7.6)	1	2
Sanitary treatments	0.17 ± 0.38 (2.5)	1	1

SD: Standard deviation.

**Table 5:** Family workforce vs. women with a job change to anyone different from dairy goat farming.

	Family Relationship with the Farmer % (*n*)			
Woman Farmer Wishing a Job Change	Wife	Sister	Mother	Without Family Relationship
Yes	26.1 (6) a	0.0 (0) b	0.0 (0) b	100.0 (3) c
No	73.9 (17) a	100.0 (4) c	100.0 (2) c	0.0 (0) b

Values with different letters in the same row are different (*p* < 0.05).

**Table 6:** Participation of women in the decision-making of dairy goat farms (%).

Decision Level	Self-Consumption	Sale of Farm Products	Loan Request	Household Costs
Main decision-taking	30.76	26.67	23.33	16.67
Her opinion is considered, but not decisive	23.08	26.67	20.00	6.67
Men and women participate equally	23.08	20.00	10.00	26.67
Do not perform this activity	23.08	26.67	46.67	50.00

## Supplementary Materials

The following supporting information can be downloaded at: https://www.mdpi.com/article/10.3390/ani12131686/s1, Survey for Data Collection.
