# Supplementary material for: The Role of Women on Dairy Goat Farms in Southern Spain
Source: Animals (Basel). 2022 Jun 30;12(13):1686. doi: 10.3390/ani12131686 (PMC9264771; doi:10.3390/ani12131686)
Supplement: Supplementary file 1 [file animals-12-01686-s001.zip › animals-1778498-Supplementary .pdf]

Survey for Data Collection

Estimado miembro de la "Asociación de criadores de la cabra Florida (ACRIFLOR)": Muchas gracias por su participación en esta encuesta anónima. Solicitamos su nombre por si necesitamos consultar alguno de los datos registrados en las bases de datos de ACRIFLOR. Con esta excepción, la encuesta es completamente anónima y bajo ninguna circunstancia se publicará información que pueda identificarle a usted o a su granja. Con estos datos, pretendemos hacer análisis estadísticos con el objetivo de publicarlos en congresos, revistas científicas y/o de divulgación. Por otra parte, nos comprometemos a mandarle, vía ACRIFLOR, los resultados de cualquier estudio que llevemos a cabo usando estos datos. Finalmente, le agradecemos su participación desinteresada en esta encuesta.

Fecha:

Nombre:

I. ASPECTOS GENERALES

1. Nombre de la Unidad de Producción:
   …………………………………………………………………………………………………
   ………
…………………………
2. Ubicación (Término Municipal, Provincia):
   ………………………………………………………………………………………..
   ………
…………………………
3. Población más cercana: ………………………………………………………………………….
   Distancia: …………………Km.
4. Sistema de explotación: Estabulado: …………………………. Semi-Estabulado:
   ………………………………………….
5. Hace uso del Campo?: Sí: …………No: ………..
   Cuál?: Olivar: ……….Dehesa: ……….Campiña-Cultivo (Vega): ……….Sierra: …………
   Espacio Natural Protegido: …………
6. Nombre del Encuestado:
   ………
   Sexo: …………… Edad: …………….años.
7. Condición del encuestado: Propietario: ……………………Encargado: ……………Ambos:
   ……………

8. Nombre del Propietario:

……

¿Aparece como Titular?: Sí: ………………No: ………………

En caso de "NO": ¿Quién es el Titular?:

………………………………………………………………………………………

Relación con el encuestado:

………………………………………………………………………………………………………

9. En caso de que el encuestado no sea el Propietario, preguntar adicionalmente datos del propietario y/o Titular:

**Propietario**	**Titular**
Nombre:	Nombre:
Edad:	Edad:
Sexo:	Sexo:
Ocupación:	Ocupación:

10. ¿Trabaja el propietario como ganadero?: Sí: …………… No: ……………

11. Número de personas que componen la Unidad familiar del ganadero:

    ……………………………………..

12. Antigüedad de la explotación:

    ……………………………………………………………………………………………….

    Tipo de empresa: Familiar: ……….., Individual: …………, Sociedad: ……….., Cooperativa: ……., Autónomo: ….…………., Comunidad de bienes: ………………

    ¿Vive el ganadero en la Unidad de Producción?: Sí: ……………, No: ……………

    En caso de NO, distancia de su casa a la Unidad de Producción: ……………….Km

13. Nivel de estudios del ganadero:

    ……………………………………………………………………………………….

14. Cursos de formación reciente del ganadero: No: ……………… Sí: …………………

    En caso de "SI", especifique:

…………………………………………………………………………………………..

……

……………………

15. ¿Le gustaría que sus hijos continuaran con la actividad ganadera?: No: ………………

    Sí: ………………

    ¿Por qué?:

………

……

……………………..

16. ¿Le gustaría que sus hijos estudiaran o hicieran otra actividad distinta de la ganadera?:

    No: ……………. Sí: ……………… ¿Por qué?:

…………………………………………………………………………….

………………………………………………………………………………………………
……..

17. ¿Recibe asistencia técnica?: Sí: ………………..No: ………………..

    Si la respuesta es SI:

ÁREA	FRECUENCIA	TIPO DE TÉCNICO	SEXO	INSTITUCIÓN	Coste (€)
Salud Animal					
Reproducción					
Nutrición					
Manejo de Pastos					
Genética					
Registros					
Otra ………………………					

II. CARACTERISTICAS DE LA MUJER

1. ¿Trabaja alguna mujer en la explotación?: No: …………. Sí: ……………..
2. Relación con el propietario. Esposa: ………………… Hija: ………………. Empleada: …………………
3. Otros: ……….
4. Edad: …………………………años
5. Nivel de Formación: …………………………………………………………………………………………… …….

………………

6. ¿Es propietaria de la explotación?: ……………………………………………………………………………….

7. ¿Desde qué año lleva trabajando con caprinos?:
   …………………………………………………………......................
8. ¿Por qué razón trabaja en la explotación?:
   …………………………………………..…………………………………
   ……
   ………………………
9. ¿Tiene hijos?: No: ………. Sí: ………. Edad de cada uno: 1º………., 2º…………..,
   3º…….……, 4º……….
   Más……

…………………..

10. Lugar de residencia:
    ………………………………………….………………………………………………………………
    ………
    ……………….
11. ¿Percibe un sueldo?: No: …………… Sí: ……………. Cantidad (Euros):
    ……………….
    ¿Cotiza en la Seguridad Social?: No: ………. Sí: ………….

12. Labores que realiza en la explotación a diario:

**Labores dentro de la explotación**	**Horas de Dedicación**	**Labores fuera de la explotación**	**Horas de dedicación**
Ordeño		Tareas del Hogar	
Limpieza de nave		Estudios	
Pastoreo		Trabajo	
Alimentación		Descanso	
Tratamientos sanitarios		Otros:	
Elaboración de queso (actualmente)			
Elaboración de queso (anteriormente)			
Tiene permiso para elab. Queso			
Otros:			

13. ¿Le gustaría realiza otra actividad?: No: ……….. Sí: ………… ¿Por qué?:
    ....
    ……

……………….…..

14. ¿Dejaría el negocio de las cabras?: No: ………. Sí: …………. ¿Por qué?:
    ....

……
………………..….

……
…………………..

15. ¿Quiere invertir más en la producción caprina?: No: ……….. Sí: ………… ¿Por qué?.............
……
……………………
……
…………………..

16. ¿Deberían sus hijos continuar con la actividad ganadera?: No: ……….. Sí: ……………
¿Por qué?:………
……………
……
……………………..

17. ¿Deberían sus hijos estudiar o hacer algo fuera de la actividad ganadera? No: ………. Sí: …….. ¿Por qué?:
……
…..
……
……………………..

18. Toma de decisiones: **más**: **+, menos: -, ninguno: /, Igual:=. Hombre: H, Mujer: M.**

	**Actividad**	**Nivel de Decisión**		**Comentarios**
		**H**	**M**	
**Producción ganadera**	Autoconsumo			
	Venta			
	Inicio de una actividad fuera de la UP			
	Inicio de otra actividad dentro de la UP			
**Económico**	Solicitud de Préstamo			
	Inversión			
	Gastos del hogar			
	Gastos en Educación			

	Gastos en salud			
	Desarrollo de la Ganadería			
**Recreación**	Salidas de paseo			
	Vacaciones			
	Actividades extraescolares			

III. ESTRUCTURA DE LA MANO DE OBRA

1. Estructura de la mano de obra (Nº de trabajadores):
   Familiares: …………. Remunerados?: Si: ………. ¿Cuántos?:…………No: ……..
¿Cuántos?: ....
   Fijos: .... Salario: ……………………………………………. Dedicación:
……………………………………
   Eventuales: .... Salario: ……………………………………………. Dedicación:
……………………………………

2. Distribución de actividades: Tipo de trabajador: Familiar, fijo, eventual.
   Actividad: Ordeñador, tractorista, quesero, etc.

TIPO DE TRABAJADOR	SEXO	ACTIVIDADES	NIVEL EDUCATIVO	TIEMPO (meses)

IV. DATOS GENERALES DE LA UNIDAD DE PRODUCCIÓN

1. Estructura del rebaño.
   1.1. Inventario animal:

ANIMAL	*Número*
Cabras: TOTALES<br>Paridas Ordeño Secas<br>Descarte	
Cabritos/as lechales	
Chivas reposición	

Chivas venta cría	
Machos	

1.2. Datos productivos y reproductivos:

ITEMS	**CANTIDAD**
**Producción de leche**	
Producción de leche (L/año)	
Leche/cabra/día (L)	
Lactancias Válidas (N.) (C.L.O.)	
Recuento de células somáticas	
Bacteriología	
Incidencia Mastitis	
**Datos reproductivos**	
Fertilidad (%)	
Épocas	
Técnicas reproductivas:	
Efecto Macho Melatonina	
Inseminación Artificial	
Esponjas	
**Producción de cabritos**	
Prolificidad (%)	
Mortalidad	
**Esquema de selección**	
Altas	
Bajas	
Parte de cubriciones	
Parte de nacimientos	
C.L.O	
